# The Current State of Antifungal Stewardship in Immunocompromised Populations

**DOI:** 10.3390/jof7050352

**Published:** 2021-04-30

**Authors:** William Alegria, Payal K. Patel

**Affiliations:** 1 Department of Quality, Patient Safety and Effectiveness, Stanford Health Care, 300 Pasteur Drive, Lane 134 L1C36, Stanford, CA 94305, USA; 2 Stanford Antimicrobial Safety and Sustainability Program, Stanford, CA 94305, USA; 3 Division of Infectious Diseases, Department of Internal Medicine, Ann Arbor VA Medical Center, Ann Arbor, MI 48105, USA; payalkp@med.umich.edu; 4 Division of Infectious Diseases, Department of Internal Medicine, University of Michigan, 2215 Fuller Rd, Ann Arbor, MI 48105, USA

**Keywords:** antifungal stewardship, immunocompromised, invasive fungal infections

## Abstract

Inappropriate antifungal use is prevalent and can lead to drug-resistant fungi, expose patients to adverse drug events, and increase healthcare costs. While antimicrobial stewardship programs have traditionally focused on antibiotic use, the need for targeted antifungal stewardship (AFS) intervention has garnered interest in recent years. Despite this, data on AFS in immunocompromised patient populations is limited. This paper will review the current state of AFS in this complex population and explore opportunities for multidisciplinary collaboration.

## 1. Introduction

Antimicrobial Stewardship Programs (ASPs) optimize patient outcomes through coordinated efforts aimed at improving antimicrobial prescribing [1,2,3,4]. In recent years, regulatory agencies have bolstered these efforts by mandating that hospitals implement ASPs modeled after the Centers for Disease Control and Prevention (CDC) Core Elements of Antibiotic Stewardship [5]. While interventions that address antibiotic use have long been considered a cornerstone of ASPs modeled after the core elements, targeted antifungal stewardship (AFS) interventions are less common. However, AFS programs have the potential to be highly impactful considering that 30–50% of antifungal use is inappropriate or suboptimal [6,7,8]. Antifungal overuse contributes to antimicrobial resistance and can expose patients to adverse drug events [9,10,11]. Additionally, given their high cost, the overuse of antifungals may pose an unnecessary financial burden for the healthcare system. While the ultimate drivers of inappropriate antifungal use are undoubtedly complex, a survey of European physicians highlighted that knowledge deficits may play a role [12]. Recognizing the need for more robust AFS efforts in clinical practice, the Mycoses Study Group Education and Research Consortium (MSGERC) published guidelines in 2020 that provide a framework for AFS programs [13].

While the impact of ASPs on antibiotic use has a robust body of literature, the impact of AFS programs, particularly among immunocompromised patients, is less described. The assessment of antifungal use in this population requires an understanding of host factors that predispose patients to invasive fungal infections (IFIs) and appropriate prophylaxis and management strategies. Additionally, limitations of diagnostic testing and an understanding of the complex pharmacological properties of antifungals must be considered when constructing AFS interventions. This paper will highlight the current state of AFS in immunocompromised patients, describe the limitations of traditional and non-culture-based fungal diagnostics, and highlight the importance of a multidisciplinary team with expertise in caring for patients requiring antifungals.

## 2. Antifungal Stewardship Principles

**Figure 1:**
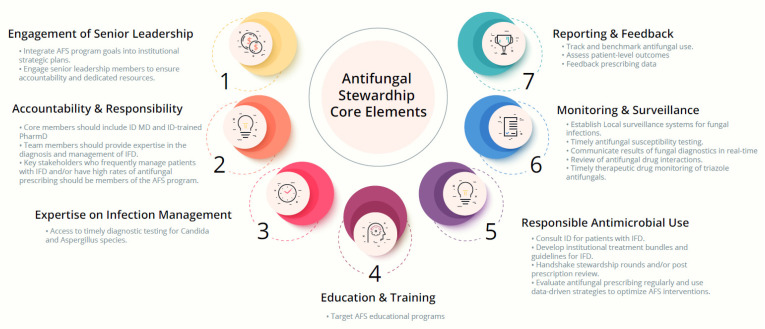
Mycoses Study Group Education and Research Consortium Antifungal Stewardship Core Elements [12].

Many of the principles of antibiotic stewardship can be incorporated into AFS programs. In fact, many of the AFS core elements proposed by the MSGERC (Figure 1) mirror the CDC core elements of antibiotic stewardship. These include (1) engagement of senior leadership, (2) accountability and responsibility, (3) expertise on infection management, (4) education and practical training, (5) actions aiming at responsible antimicrobial use, (6) monitoring and surveillance, and (7) reporting and feedback [13]. We refer readers to the MSGERC guideline for detailed review of the AFS core elements and appraisal of the available literature. Despite these similarities, AFS programs must consider the complex, heterogeneous patient populations that often require antifungal therapy. AFS interventions should also be tailored to the patient population of interest and developed in collaboration with key stakeholders within that specialty area. As part of a robust AFS program, in addition to Infectious Diseases (ID) physicians and ID-trained pharmacists, healthcare professionals with expertise in hematological malignancies, solid organ transplant (SOT), hematopoietic cell transplantation (HCT), and clinical microbiology should be included (Table 1).

Tools commonly used in general ASP interventions such as prospective audit and feedback and preauthorization may be leveraged to improve antifungal prescribing. Prospective audit and feedback engages the provider after an antibiotic is prescribed whereas preauthorization requires approval before an antimicrobial can be initiated. The advantages and disadvantages of both strategies have been described previously [1]. Finally, an assessment of baseline antifungal use and benchmarking can help identify priorities for improving antifungal prescribing within the institution. For institutions that report to the National Healthcare Safety Network (NHSN) Antimicrobial Use and Antimicrobial Resistance module, risk-adjusted antifungal use data for hematology-oncology wards may be helpful.

## 3. Antifungal Stewardship Interventions

Published AFS interventions have demonstrated reductions in antifungal consumption and antifungal drug expenditure without adversely affecting clinical outcomes [15]. Recent systematic reviews of AFS studies have highlighted that most interventions rely heavily on prospective audit and feedback and preauthorization [15,16]. Many of the studies included in these systematic reviews and those published elsewhere have focused on patients with candidemia in the intensive care unit (ICU) [2,17,18,19]. These interventions commonly involve “care bundles” or “checklists” of best practices prioritizing early detection and diagnosis, early initiation of antifungal therapy, and source control. This same concept has been applied to the management of *Staphylococcus aureus* bacteremia and has been linked to improved patient outcomes [20,21]. While these interventional studies in candidemia have demonstrated increased compliance with elements of the care bundle and reduced antifungal consumption, few have demonstrated improvements in clinical outcomes. A notable exception is a recently published single-center, quasi-experimental pre-post study that demonstrated a significant reduction in 14- and 30-day all-cause mortality after implementation of a care bundle in patients with candidemia [19].

Despite this promising data, caution should be exercised when attempting to generalize these findings to immunocompromised populations. Non-candida opportunistic yeasts, many of which are echinocandin non-susceptible, may disproportionately affect immunocompromised patients and make the application of these bundles challenging [22,23,24,25]. Additionally, clinicians should consider how host factors influence the type of fungal infections patients are at risk for and tailor treatment and prophylaxis accordingly. In our view, these nuances highlight the importance of multidisciplinary collaboration and suggests that a dedicated AFS program, in addition to existing ASP efforts, may be advantageous.

## 4. Antifungal Stewardship in Immunocompromised Populations

### 4.1. Hematology-Oncology

IFIs are associated with a high rate of morbidity and mortality in hematology-oncology patients [26]. As a result, these patients often receive varied and prolonged courses of antifungals for the treatment and prophylaxis of IFIs. Although literature in this population is scarce, antifungal use has been found to deviate from guideline and/or labeling recommendations in a substantial proportion of cases [27].

A recent study in pediatric hematology-oncology patients evaluated antifungal prescribing before and after implementation of a protocol for the management of IFIs [28]. After implementation, inadequate prescriptions as adjudicated by an external evaluator decreased by roughly 10%. The intervention also had an educational component that was associated with a significant improvement in knowledge of IFI management that persisted at 12 months. A second study in pediatric patients at a tertiary care center, inclusive of 48 hematology-oncology beds, demonstrated a reduction in antifungal Days of Therapy (DOT)/1000 patient-days after implementation of a “handshake” stewardship intervention [29]. It should be noted that this study did not restrict or require preauthorization for any antimicrobials.

A study in adult hematology patients evaluated the impact of a multi-pronged stewardship intervention over nine years [30]. The intervention included twice-weekly in-person consultation with an ID physician, telephone counseling, training sessions for prescribers at least once yearly, and the development of a clinical algorithm for management of proven or probable invasive aspergillosis. The intervention was associated with a decrease in antifungal consumption of 40% despite a stable rate of IFIs throughout the study period. AFS interventions leveraging post-prescription review of targeted antifungals at tertiary care centers, including patients admitted to hematology-oncology teams, have also demonstrated decreased antifungal consumption and proved to be a cost-effective strategy [31,32].

Guidelines for hematology-oncology patients should also be developed to address considerations for initiation of antifungal therapy, diagnostic work-up, and recommendations for consultation with ID physicians. These algorithms should incorporate evidence-based recommendations from national practice guidelines and provide options based on the availability of formulary antifungals. Education could then be provided to hematology-oncology clinicians to support guideline implementation. Prospective audit and feedback, an intervention associated with improved long-term acceptance rates, can then be used to provide clinicians feedback and assess guideline compliance [33].

Institutional guidelines can also be used to address high-risk hematology-oncology patients in whom antifungal prophylaxis has been associated with clinical benefit (e.g., acute myeloid leukemia (AML) patients receiving high-intensity induction chemotherapy) and discourage use in lower-risk patients where no clear benefit has been established [34]. These guidelines can also draw attention to contraindicated drug-drug interactions (DDIs) between chemotherapy and azole antifungals and provide alternative recommendations as needed. Embedding recommendations into existing antimicrobial use guidelines may also be more convenient for front-line clinicians. Since IFIs in this population may delay chemotherapy and have been correlated with an increased risk of death in case of remission, attempts to mitigate progression to invasive disease should be pursued [35,36]. Table 2 highlights how institutional guidelines can be leveraged to provide recommendations for antifungal use in patients with AML, acute lymphocytic leukemia (ALL), lymphoma, and multiple myeloma (MM) receiving chemotherapy.

### 4.2. Solid Organ Transplant

IFIs in SOT patients are an important cause of morbidity and mortality [37]. This, coupled with limitations in available diagnostics, may partially explain the low rates of compliance with antifungal use guidelines among transplant physicians [38]. Literature evaluating the impact of targeted AFS interventions among SOT patients is also limited.

Building on the concept of an ASP “timeout” which has been evaluated for ASPs and supported by the CDC [5], Mularoni et al. performed a multifaceted intervention using a compulsory antifungal “time-out” coupled with ID physician review at 72 h. This intervention was associated with a discontinuation rate of 48% among SOT patients [39]. The “time-out” prompted a clinical reassessment of the patient, as well as a review of all available microbiological and radiological data. The intervention was associated with an improvement in guideline-concordant antifungal selection, dosing, and duration. Academic detailing rounds led by ID-trained pharmacists and transplant ID physicians has also been proposed as an intervention to improve concordance with AFS recommendations in SOT patients without negatively impacting outcomes [40]. Education to transplant physicians on antifungal prescribing should also be considered as an intervention to improve antifungal utilization. Martin-Gutierrez et al. described a comprehensive educational program with long-term maintenance focused on antifungal use at a referral center for SOT patients. Over a 9-year period, the intervention was associated with a significant and long-lasting reduction in antifungal consumption without negative impacts on mortality [41].

## 5. Fungal Diagnostic Testing

Many of the traditional fungal diagnostics are neither sensitive nor specific for IFIs and make establishing a definitive diagnosis challenging [42,43,44]. Histopathology, the gold standard for proven IFI, may also be difficult or impossible to obtain in critically ill immunocompromised patients [45]. In the absence of histopathology, distinguishing colonization from infection can be challenging. Despite improvements in the last few decades with the advent of non-culture-based diagnostics, data suggest that missed diagnoses still occur in immunocompromised patients [46]. Antifungal prescribing must therefore balance the consequences of delayed or missed diagnoses with the deleterious consequences of antifungal overuse.

### 5.1. Non-Culture Based Diagnostics

Several non-culture-based tests (NCBT) are used clinically in the diagnosis of IFIs. These include (1→3)-β-d-glucan (BDG), galactomannan, *Aspergillus* polymerase chain reaction (PCR), mannan, and antimannan [44,47]. Along with clinical and radiological findings, the results of NCBTs may be used to initiate antifungal therapy in hematology and HCT recipients [48,49]. The interpretation of results should be made carefully and in the context of the patients’ immune status, considering the prevalence of the disease in the population and any other factors that may impact the rate of false positives or false negatives [50,51]. While data to support the role of NCBTs such as BDG in bedside AFS interventions is limited, a pre-post study at a large SOT and oncology referral center in Spain demonstrated an improvement in the adequacy of antifungal use and reduction in drug expenditure without negative impacts on mortality [52]. While future studies are needed, NCBTs may play a role in AFS efforts in the future.

### 5.2. Diagnostic Stewardship

Despite their promising role in the diagnosis of IFIs, the lack of specificity of NCBTs may drive unnecessary antifungal use. In a study of non-neutropenic patients who were ordered a BDG at an academic medical center, a retrospective review by 2 independent ID physicians revealed that orders were inappropriate in nearly 50% of cases [53]. Similarly, in a cohort of 470 patients, BDG and galactomannan orders were inappropriate in roughly 75% of cases. On multivariate logistic regression, admission to transplant medicine, lack of consultation with ID, and absence of predisposing factors were associated with the inappropriate ordering of NCBTs [54]. The complexities of NCBTs reinforces the need for multidisciplinary care of patients with suspected IFIs, including clinical microbiologists who can provide clinicians with an in-depth understanding of their performance and interpretation [55].

## 6. Multidisciplinary Collaboration

### 6.1. Clinical Pharmacists

In addition to specialty physicians, clinical pharmacists are critical allies in AFS efforts [55].

In one study, contraindicated DDIs were reported in nearly 25% of inpatients prescribed mold-active triazoles [56]. Many of the novel therapeutics in hematology-oncology and drugs used for maintenance of immunosuppression in SOT patients also have clinically actionable DDIs with triazoles [57,58]. These interactions may require the monitoring of serum levels and/or dose adjustments. Clinical pharmacists are well-positioned in such instances to provide consultative services to clinicians to help ensure the safety and efficacy of the regimens prescribed.

Many of the antifungals used for the treatment and prevention of IFIs also have complex pharmacokinetics that leads to inter-and intra-patient variability in drug exposure [59,60]. This may be further compounded in immunocompromised patients who experience complications of treatment such as graft versus host disease or severe mucositis [61]. This variability, coupled with known exposure-response targets associated with efficacy and safety, make therapeutic drug monitoring (TDM) necessary for many of the triazoles [60,62,63]. Clinical pharmacists should be available to provide recommendations on the appropriate timing of TDM, aid in the interpretation of serum drug levels, and provide recommendations for dose adjustments as needed. Also, pharmacists can help monitor for ADEs, aid in the appropriate and timely conversion of enteral therapy and facilitate transitions of care for costly antifungals.

### 6.2. Clinical Microbiology and Laboratory Collaboration

Clinical microbiologists are key collaborators of a robust AFS program. In addition to helping with the implementation and interpretation of traditional and NCBTs, they are critical in the validation of antifungal susceptibility testing (AST). They are uniquely aware of the limitations of AST and may be the only members of the team with in-depth knowledge of interlaboratory variability among testing platforms or methodologies.

Laboratory collaboration is also key to ensure the timely in-house availability of antifungal drug levels as these have been associated with reduced time to drug concentration results and time to therapeutic serum concentrations [64]. In collaboration with other members of the AFS program, in-house tests can be prioritized based on patient population and clinical need. Since immunocompromised patients are disproportionately affected by IFIs and subtherapeutic concentrations have been associated with higher patient mortality, centers should carefully consider in-house versus “send-out” antifungal TDM and prioritize the former whenever possible [64].

## 7. Conclusions

Although limited data exist regarding effective AFS interventions in immunocompromised patients, published strategies to date are reassuring and should motivate future research in this space. It is not surprising that many of these interventions mirror principles of general ASPs (e.g., PAF, preauthorization, handshake stewardship, education, etc.). When the limited number of antifungal drugs available is considered, the argument for expanded AFS is compelling. As new antifungal drugs will be approved in the coming years, the need for a dedicated team of clinicians to steward their use will only increase [65]. Despite this need, a recent survey of Society for Healthcare Epidemiology of America Research Network Hospitals revealed that institutional guidelines for the management of IFIs are only available in roughly 50% of centers [66]. This is more striking when one considers that 60% of survey respondents indicated being a part of a team with greater than five members. In our view, this supports the need for more comprehensive AFS efforts even among large ASPs. Additional areas of improvement should include diagnostic stewardship and increase interdisciplinary collaboration. Given the unique vulnerabilities of immunocompromised patients, we challenge ASPs to build upon their current efforts with antibiotics to preserve current antifungals, limit unnecessary drug toxicity, and improve patient outcomes.

## Figures and Tables

**Table 1:** Example of Incorporating Antifungal Stewardship into Prophylaxis for Hematology/Oncology Patients [14].

	Antifungal	Antibacterial	Antiviral	PJP
General Considerations	ANC < 500 cells/mm^3^ for >7 days Mucositis (increased candidiasis risk) >10% risk of candidiasis Consider mold-active prophylaxis when >6–8% risk of aspergillosis	ANC < 500 cells/mm^3^ for >7 days Weigh risks of prolonged antimicrobial exposure	HSV or VZV seropositive Prior HSV or VZV episode T-cell suppression Prolonged neutropeniaMucositis	>3.5% risk of developing PJP T-cell suppression (especially CD4 <200 cells/mm^3^)
Utility	Reduce risk of fungal infection and related mortality	Reduce risk of bacteremia and fever Potential mortality benefit	Reduce risk of viral reactivation	Reduce risk of PJP infection and related mortality
Agents Preferred	Fluconazole (candida prophylaxis only) Posaconazole (mold-active prophylaxis)	Levofloxacin	Acyclovir	TMP/SMX
Alternative(s)	If drug interaction, intolerance, or contraindication (consider spectrum indicated): caspofungin, isavuconazole, liposomal amphotericin B, voriconazole	If intolerance, contraindication, or allergy to fluoroquinolone: cefpodoxime	If patient preference: famciclovir, valacyclovir	If drug interaction, intolerance, allergy, or contraindication to TMP/SMX: atovaquone, dapsone, inhaled pentamidine
AML Induction	Posaconazole during neutropenia	Consider during neutropenia	During treatment course	Consider if purine analog
Consolidation or low-intensity treatment	Consider posaconazole if ANC < 500 cells/mm^3^ for >7 days	No routine prophylaxis		
ALL Induction through maintenance	Fluconazole or caspofungin during neutropenia	Consider during neutropenia	During treatment course	During treatment course
Blinatumomab (for relapsed/refractory ALL)	Consider mold-active prophylaxis based on duration and depth of neutropenia	No routine prophylaxis	Consider during treatment course	Consider during treatment course
Lymphoma Most regimens	No routine prophylaxis	No routine prophylaxis	Consider during treatment course	No routine prophylaxis, consider if prolonged CD4 < 200 cells/mm^3^
Intensive chemotherapy	Consider fluconazole during neutropenia	Consider during neutropenia		
MT-R for PCNSL	No routine prophylaxis	No routine prophylaxis		During treatment course (avoid TMP/SMX during HD-MTX)
Multiple Myeloma Proteasome inhibitors	No routine prophylaxis	No routine prophylaxis	During treatment course	No routine prophylaxis
Daratumumab			During treatment course and 3 months after	
Intensive chemotherapy	Consider fluconazole during neutropenia	Consider during neutropenia	Consider during treatment course	

**Abbreviations:** ALL: acute lymphoblastic leukemia; AML: acute myeloid leukemia; ANC: absolute neutrophil count; CD4: cluster of differentiation 4; HD-MTX: high-dose methotrexate; MT-R: high-dose methotrexate with temozolomide and rituximab; HSV: herpes simplex viruses; PCNSL: primary central nervous system lymphoma; PJP: pneumocystis jirovecii pneumonia; TMP/SMX: trimethoprim/sulfamethoxazole: VZV: varicella-zoster virus.

**Table 2:** Core members of an Antifungal Stewardship Program.

Core Members	Potential Contributions
ID physicians	- Diagnostic testing/stewardship - Antifungal drug selection and assessment of treatment response - Assessment of antifungal related ADEs - Development of clinical guidelines and pathways aimed at optimizing antifungal use
ID-trained pharmacists	- Assessment of baseline antifungal use and benchmarking - Education to clinicians, including physicians and clinical pharmacists, on appropriate antifungal use - Antifungal formulary management - Development of clinical guidelines and pathways aimed at optimizing antifungal use
Primary Team Clinicians - HCT - Hematology/ - Oncology - SOT	- Risk-stratification and indications for antifungal prophylaxis based on patient-specific factors: - Type of transplant, conditioning regimen, presence of graft versus host disease, etc. - Underlying malignancy and response to chemotherapy - Type of transplant, induction, maintenance, and anti-rejection immunosuppression in SOT recipients - Management of DDIs - Immunosuppression in HCT and SOT recipients (e.g., calcineurin inhibitors) - Traditional chemotherapy (e.g., vinca alkaloids) - Novel targeted therapies (e.g., venetoclax) - Antifungal prescribing for treatment of IFIs
Clinical Pharmacists	- Screen and manage DDIs - Facilitate transitions of care for high-cost antifungals (e.g., mold-active triazoles and flucytosine) - Interpretation of therapeutic drug monitoring (e.g., voriconazole and posaconazole) - Assessment of antifungal related ADEs
Clinical Microbiologists	- Implement and interpret fungal diagnostics (e.g., traditional and non-cultured based tests) - Implement and validate antifungal susceptibility testing

**Abbreviations:** ADE: adverse drug event; DDI: drug-drug interaction; HCT: hematopoietic cell transplantation; IFI: invasive fungal infection; SOT: solid organ transplantation.

## Abbreviations

AFS	antifungal stewardship
ID	infectious diseases
FD	invasive fungal disease
MD	Medical Doctor
PharmD	Doctor of Pharmacy
